# Long-Term Heavy Metal Retention by Mangroves and Effect on Its Growth: A Field Inventory and Scenario Simulation

**DOI:** 10.3390/ijerph17239131

**Published:** 2020-12-07

**Authors:** Anh Nguyen, Otto Richter, Bao V.Q. Le, Nguyen Thi Kim Phuong, Kim Chi Dinh

**Affiliations:** 1 Institute for Environment and Resources, Vietnam National University Ho Chi Minh City, 142 To Hien Thanh, District 10, Ho Chi Minh City 72506, Vietnam; levuquocbao.env@gmail.com (B.V.Q.L.); dkchi2012@gmail.com (K.C.D.); 2 Institute of Geoecology, Technical University of Braunschweig, 38106 Braunschweig, Germany; o.richter@tu-bs.de; 3 National Institute of Applied Mechanics and Informatics, Vietnam Academy of Science and Technology, 291 Dien Bien Phu, District 3, Ho Chi Minh City 722007, Vietnam; nguyenthikimp@yahoo.ca

**Keywords:** mangroves, mangrove modelling, *Rhizophora apiculata*, phytoremediation, heavy metal pollution

## Abstract

The ability of mangroves in taking up and storing heavy metal (HM) helps in reducing HM pollution. However, HMs likewise adversely affect the growth of mangroves. We assess the effects of the long-term soil HMs enrichment on the growth of *Rhizophora apiculata* forest in the Can Gio Mangrove Forest (Southern Vietnam) in different environmental conditions of soil salinity, ground elevation, and tree density based on a novel set of measured data. These data were analyzed and were used to calibrate and validate for a tree growth model with influencing factors salinity, elevation, tree density, and heavy metals content. Three scenario simulations were performed to predict the mangrove dynamics under different levels of heavy metal pollution in combined environmental conditions of salinity and elevation. Simulation results show the decline of total forest biomass from 1,750,000 tons (baseline scenario with no HM pollution) down to 850,000 tons and 350,000 tons for the current HM pollution and double HM pollution scenarios, respectively. Both data analysis and simulations have shown that although mangroves can assist in reducing HM pollution, the quality and health of this ecosystem will be severely affected if the environment is excessively polluted. In addition, a data-and-model driven management tool is devised for the sustainable management of the mangrove environmental resources.

## 1. Introduction

Mangrove ecosystems are adversely affected by global and local stresses. Environmental stress, such as pollution, and especially heavy metal (HM) pollution, has been extensively addressed worldwide because of its adverse effects on ecosystems and human health. Excess of sedimentary HM concentrations constitute a potential risk of ecosystem health. It is a challenge to devise techniques for the reduction and cleanup of pollutants in the highly dynamic environment of mangrove forests.

Naturally, mangrove trees have the ability for storing metals, transferring these elements from the sediment and concentrating them in their tissues [1]. They act as a biological uptake medium and are a convenient means for phytoremediation [2]. Mangroves remove metals through absorption, cation exchange, filtration, and chemical changes through root accumulation processes [3]. There are many cases in which wetland plants (mangroves) are utilized for removal of pollutants, including metals. This functionality of mangroves has been studied and proven to be a good practical method to treat pollutants [4,5,6,7,8,9,10].

However, excessive heavy metal pollution causes a series of physiological and biochemical alterations affecting the growth, metabolism, and cell structure of plants [11] with the implication of potential ecological system risks [12,13]. Plants may alter the speciation of metals and may also suffer toxic effects as a result of accumulating them. Plant’s behaviors when being stressed by heavy metals were examined by greenhouse experimental studies, which applied for young mangroves, including oxidative stress (malondialdehyde (MDA) [14]), osmotic stress [15], inhibition of photosynthesis, inhibition of growth, and increased mortality [16].

To assess plants stress by heavy metals in the field, extensive empirical observations are required. However, in field observations, spatial heterogeneity concerning ecotope composition, initial contaminant levels and other factors make a consistent data analysis difficult. This holds especially true for the situation in large ecosystems with spatially varying environmental and pollutant conditions such as in mangrove areas. The structure of mangrove forests, their health and their ecosystem functions are strongly influenced by the factors resources (nutrients), regulators (soil salinity, pollutants) and hydro-regimes (duration, frequency and depth of inundation) and their mutual interactions [17,18,19,20,21].

This problem can be overcome by employing mathematical modelling tools which incorporate different factors into an integrated system capable of predicting ecosystem behavior under multiple stressors. While field inventories are resource-demanding, advances in mathematical modeling are now providing globally coterminous datasets at high spatial resolutions. There are quite a few different modelling approaches. Individual-based models (IBMs) for mangroves were developed for the assessment of the effects of natural threats and applied to the management of mangrove forest stability for coastal protection using a combination of windthrow models [22,23,24]. Quantitative statistical and a qualitative categorical ranking approaches were also used to identify risk factors for mangrove lost in Kenya (Rideout et al. [25]). Box models were applied for establishing mass balances for heavy metals. In the paper of Mandal et al. [26] the up-taken as budget for the Sundarbans mangroves was calculated. A phytoremediation model for the mangrove species *Rhizophora apiculata* was developed [27] coupling growth dynamics of plant organs with Cr uptake taking into account adverse effects of Cr in the plant. Also, a phytoremediation model at catchment scale combining hydrodynamics, Cr transport, and fate of chromium in the soil-plant domain [28] was realized by integration of an environmental fate model into the hydrodynamic model Delft3D and applied to the Thi Vai catchment in Vietnam.

The emerging problems in research on the mangrove retention of HM in relation to its growth concern the tradeoff between the phytoremediation function and the adverse effects of HM. In this context, we focus on *R. apiculata* forest in the Can Gio area, southern Vietnam. Previous studies in this area mainly focused on the potential of biological effects and ecological risk of heavy metal accumulation in the sediments. Due to the important role of ecosystem services provided by the Can Gio Mangrove [29,30], it was said that more attention should be paid on long-term trends of HMs and the complex interplay between hydrodynamics, anthropogenic stressors and HM concentrations, as well as ecosystem health [29,31,32]. Thus, our study aims to:

(i) Review and re-evaluate HMs contamination degrees in mangrove soil;
(ii) assess the effects of the long-term HM pollution on the growth of *R. apiculata* in the interactions with the effects of the natural factors soil salinity, ground elevation and tree density; and
(iii) perform scenario simulations of mangrove dynamics under different levels of HM pollution and other natural stressors.

Establishment of reasonable model parameters is a major issue because of the lack of appropriately designed long term field experiments. In our study area we are in a favorable situation since the mangroves were planted and their age were recorded and the data on elevation and salinity are available. So this situation constitutes, though not planned in the first place, an “experimental design” suitable for the purpose of parameter estimation. To this end, the set of parameters for growth response functions of a previously developed mangrove dynamic model CGMM [33] were estimated based on the measured data in 208 sites in the Can Gio mangrove forest.

## 2. Materials and Methods

### 2.1. The Can Gio Mangrove Forest

**Figure 1:**
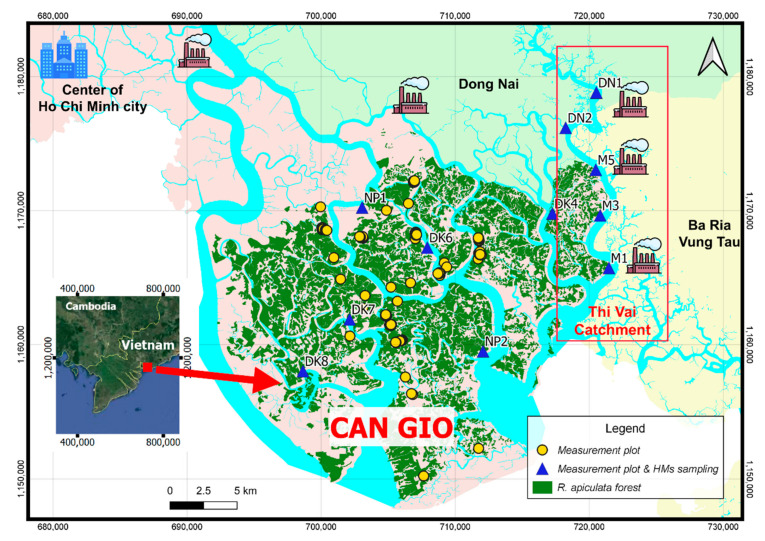
The Can Gio mangrove area and the mensuration plots. Thi Vai catchment (inside the red frame) is affected by the industrial activities on the East bank and the Can Gio area is directly influenced by activities from the center of Ho Chi Minh city. The round yellow points are the measurement plots without taking samples. The triangle blue points are the measuring plus sampling plots for heavy metals (HMs) content determination.

The Can Gio Mangrove Forest (in the South of Vietnam) is located ca. 40 km off the biggest industrial city in Vietnam—Ho Chi Minh City (Figure 1). This forest is one of the most extensive rehabilitated mangrove forests in the world. The planted mangrove species *Rhizophora apiculata* Blume covers most of the forest. *Rhizophora apiculata* in this forest has been growing over 40 years since 1978. The structure of this mangrove forest is remarkably diverse and provides an excellent case study for research. Regeneration is assisted by planting of seedlings/saplings, but the trees also commence natural generative reproduction and contribute to the propagule input. The silvicultural practice used to consist of thinning events which removed competing dominant trees. Since this forest was nominated as “Biosphere Reserve” by UNESCO in 2000, the forest was strictly protected, and the activity of forest thinning was inhibited. The mangrove ecosystem in this area was said providing not only various goods and services to local people such as timber, seedlings, medicines, but also foods because variety of marine organisms live within and around this mangrove, e.g., shrimp, fish, snail, or crab [30].

This mangrove area is subject to asymmetric semidiurnal tidal regimes and typical tropical monsoon climate with two distinct seasons, dry season (extends from November to next April) and rainy season (from May to October). Mean water salinity varies from 12 to 30 part per thousand (ppt). Salinity changes by season, highest salinity occurs in dry season and reaches the value of more than 30 ppt in March and April. Flooding frequency and elevation of the study area affect primary production and species distribution in Can Gio [34,35]. van Loon et al. [36] classified Can Gio mangroves into five classes based on tidal regimes: High tidal areas (no mangroves observed), medium high tidal areas (*Avicennia* spp., *Sonneratia*), normal high tidal areas (*Rhizophora* spp., *Ceriops*, *Brugueira*), spring high tidal areas (*Lumnitzera*, *Brugueira*, *Acrostichum aureum*), and equinoctial tidal areas (*Ceriops* spp., *Phoenix paludosa*).

The area has been found being polluted by HMs. Our previous publication [37] reported high concentrations of Cr, Cu, and Ni in the soils and tree organs among the 18 elements extracted from samples in the 11 locations as shown in Figure 1 (the triangle blue points). Costa-Böddeker et al. [31,32,38] also gave evidences of the long-term pollution of these HMs in this area. Mean sedimentation rates calculated from the sediment cores are remarkably high (9.2 cm year^−1^ upstream and 4.7 cm year^−1^ downstream) due to the rapid urbanization and industrialization in the recent two decades as was reported by Costa-Böddeker et al. [32,38]. However, natural processes such as tidal currents and heavy rainfall also play a key role on sediment dynamics in this area [39]. These high sediment fluxes were also associated with increasing HMs contaminants in the area.

### 2.2. Sampling Positions, Samples Preparation, and Analysis

A comprehensive field investigation was conducted in 2018 for 208 sampling plots of 10 × 10 square meters (the points in Figure 1) located in the area. The key growth driving factors inundation frequency (as expressed via ground elevation) and soil water salinity were determined for each plot. In addition, measurements of the mangrove tree heights (*H*), diameters at breast height (*dbh*), and tree density were conducted. The age of the forest stands is well known because it is a planted monospecific forest mostly covered by *R. apiculata*. The monospecific structure and the even age favor the insight into species–environment interactions. Thus, this monospecific planted forest plays an important role as a long-term experiment allowing the study of influences of environmental drivers on tree development.

The salinity (in part per thousand, ppt) of pore water in the soils was determined using an Atago refractometer. After extracting a drop of interstitial water from sediments, pore-water from the sediments was collected using a syringe. In the 11 out of the 208 sampling plots (marked by triangle blue points in Figure 1) HMs concentrations in soils, roots and leaves were determined. These plots are located at the same positions as the 2013-sampling plots from our previous work (Nguyen et al. [37]).

Samples of soils (0–30 cm depth) were collected and covered carefully with plastic scrapers. Soil samples were homogenized in an agate mortar. Every 5 g of the oven dried homogenized soil samples were directly digested with aqua regia. The concentrations of HMs (Cr, Cu, Fe, Ni) in the aqua regia extracts were determined with an ICP OES (Optima 2100 DV, PerkinElmer, USA). The aqua regia extraction for Fe is according to the ISO 12914. Up to 0.5 g of sample was placed in a Teflon vessel with 12 mL of aqua regia (37% HCl:70% HNO_3_ (3:1) mixture). The vessels were heated in a microwave apparatus (MARS 6-CEM, USA) up to 180 °C within 6 min and remained at 180 °C for 10 min. For the other HMs, the extraction was based on the ISO 11466.3. Up to 5.0 g of sample was placed in a 250 mL Pyrex beaker with 40 mL of aqua regia (37% HCl:70% HNO_3_ (3:1) mixture). The beaker was heated at 130 °C for about 30 min and evaporated almost to dryness. The residue was re-suspended using HNO_3_ 1.6 M. After centrifuging the supernatant was diluted to 25 mL [40,41].

Tree roots and leaves were collected on one adult tree in each of the 11 sampling plots. The samples were analyzed at the Hochiminh city Institute of Resource Geography, Vietnam Academy of Science and Technology, Vietnam. All plant samples were rinsed with pure water to eliminate residues of sea water and soil attached. Representative parts of the plant samples and aliquots of the soil samples were air dried at room temperature. The cut root samples and leaves were milled under addition of liquid nitrogen. Aliquots of 1 g were dried in an oven at 105 °C according to DIN EN 212 (2003) until constant mass was reached. The dry mass was used as base for the calculations of the concentrations of elements. Aliquots of 3 g to 10 g of the air dried and milled plant samples were reduced to ashes according to DIN 19684-3 (2000) at 550 °C and the residues were digested with aqua regia according to DIN EN 13346 (2000).

### 2.3. Tree Growth Model

The structure and conceptual basis of the growth model are based on the classical Botkin model [42]. The model equations are given by the differential equation for the tree diameter (*dbh*) and the relationship between diameter and tree height (*H*).

#### 2.3.1. Tree Growth Equation

The growth equation is expressed in the Equations (1)–(4) [42].

(1) $$\frac{d\left(dbh\right)}{dt}=G_{opt}\times \frac{dbh\cdot \left(1-dbh\cdot \frac{H}{D_{max}\cdot H_{max}}\right)}{2b_{1}+3b_{2}\cdot dbh-4b_{3}\cdot dbh^{2}}\times MUL,$$

(2) $$H=b_{1}+b_{2}\cdot dbh-b_{3}\cdot dbh^{2}$$

(3) $$b_{2}=\frac{2\left(H_{max}-b_{1}\right)}{D_{max}}$$

(4) $$b_{3}=\frac{H_{max}-b_{1}}{D_{max}^{2}}$$

(5) $$Biom=a_{1}\cdot dbh^{c_{1}}$$

where: *dbh* is the diameter at breast height in cm; *H* is the tree height in cm; $H_{max}$ and $D_{max}$ are the species specific maximum values of tree height and tree diameter; $b_{1}$, $b_{2}$, and $b_{3}$ are the parameters characterizing the relationship between *H* and *dbh*; $G_{opt}$ is the species specific growth rate under optimal environmental condition; $a_{1}$ and $c_{1}$ are scaling parameters for calculating tree biomass Biom from *dbh* value; *MUL* is the growth multiplier depending on environmental conditions and tree competition. It takes values between 0 and 1. In this study, *MUL* comprises of the influencing factors salinity, ground elevation, tree density and heavy metal pollutants.

Here we omit the inner physiological differentiation among trees of the same species and assume that mangrove structure and productivity are governed by environmental factors and by their own interactions (competition). Thus, at a given age, trees of the same species will attain the same size under optimal conditions for tree growth.

The form of multiplier function for salinity and density factors is

(6) $$g_{i}\left(x\right)=\frac{1-a0_{i}}{1+e^{-d_{i}\left(tr_{i}-x_{i}\right)}}+a0_{i}.$$

This multiplier function is determined by the threshold value $tr_{i}$, the slope $d_{i}$ and the asymptotic value $a0_{i}$ ensuring that the growth rate remains at a small level even at high values of salinity or tree density. The index *i* refers to the salinity and tree density, respectively. This approach is motivated by the salinity dependence of the growth of mangroves as published by Chen and Twilley [22] or by Berger and Hildenbrandt [24].

Elevation can be considered as the factor that reflects the frequency and duration of tidal flooding, thus, instead of considering two factors that influence tree development (tidal frequency and duration) we integrated both into only one factor (elevation). The multiplier function for elevation is (from Anh [33]).

(7) fel=amax−a1e1−e−el−elminel1α+a1ee−el−elminel2β+a2e1−e−el−elminel2β.

It is conceived such that it takes on the minimal value $a_{1e}$ at low elevations and the minimal value $a_{2e}$ at high elevations, exhibiting an optimal value or a plateau of optimal values in between. It is set equal to zero below the minimum elevation $el_{min}$.

The form of multiplier function for pollutant is

(8) $$P\left(x\right)=\left[1-e^{-{\left(\frac{x}{th1}\right)}^{\alpha 1}}\right]\cdot e^{-{\left(\frac{x}{th2}\right)}^{\alpha 2}}.$$

This function for pollutants takes into account hormetic growth stimulation. Hormetic growth stimulation stands for stimulatory effects at low doses and was reported for Cd, Cr, Al, Se, and Pb [43,44]. The form of this response curve is determined by the threshold value $th_{i},\;\left(i=1,2\right)$ and the form factors α1, and α2. Below threshold value $th_{1}$ of HM concentration tree growth rate increases, above threshold value $th_{2}$ it decreases.

#### 2.3.2. Calculation of Tree Growth Rate G

Under influences of environmental factors, the actual value of the growth rate *G* of a tree is always smaller than the optimum $G_{opt}$ value and is reduced by multipliers *MUL* reflecting the influence of environmental conditions: $G=G_{opt}\cdot MUL$.

In order to analyze relationships between influencing factors and tree development, values of tree growth rate *G* must be known. Analysis of tree development based on *G* values is more precise than based on size of trees (diameter and height) since *G* averages the growth of a tree taking into account also the factor tree age and influencing factors (MUL).

Integration of Equation (1) yields an expression of *G* as a function of the diameter at the time of planting, $dbh_{0}$, and at the time of measuring (age at the time of measurement), $dbh_{age}$ [33]

(9) $$G=\frac{2\cdot H_{max}\left[\int \frac{b_{1}+a\left(3e^{x}-2e^{2x}\right)}{a+b_{1}-e^{x}\left(b_{1}+a\left(2e^{x}-e^{2x}\right)\right)}dx-C\right]}{t},$$

where $a=H_{max}-b_{1}$, $x0=ln\left(dbh_{0}/D_{max}\right)$, $x1=ln\left(dbh_{age}/D_{max}\right)$, *C* is an integration constant.

### 2.4. Data Analysis and Model Parameters Estimation

#### 2.4.1. Data Analysis

HM levels were evaluated by reference to the Enrichment Factor (EF) [45,46]. This factor normalizes the element concentration with respect to a reference element which is not expected to be enriched from anthropogenic sources. Therefore, our assessment was based on the *EF* index. We used iron (Fe) as reference element. The *EF* is calculated as follow (Equation (10)):

(10) $$EF=\frac{\left(\frac{Me}{Fe}\right)Sample}{\left(\frac{Me}{Fe}\right)Background},$$

where $\left(\frac{Me}{Fe}\right)Sample$ is the ratio of the concentrations of the element to Fe in the sample and $\left(\frac{Me}{Fe}\right)Background$ is the ratio of the concentrations of the reference material (background values). *EF* values lower than 1.5 suggest that the element is derived mainly from natural sources, *EF* values higher than 1.5 suggest anthropogenic sources [47] whereas *EF* > 5 shows a significant HM enrichment [46].

Continental shale (CS) averages [48] were used as background values for the calculations of this index. The use of CS instead of regional levels as background values could lead to a conflicting pollution classification which may hamper the assessment of environmental impacts in this region [38,49]. Unfortunately, it was not possible to get data before the period of anthropogenic pressures which considerably modified this area.

#### 2.4.2. Model Parameter Estimation

The growth model as explained in the Section 2.3 needs to be parameterized for the mangrove species *R. apiculata*. This parameterization is based on the 208 measured data of tree stem heights and stem diameters at breast height with age ranging from 19 to 40 years.

Among the analyzed 208 data plots, 65 plots were chosen for model parameter estimation. The locations were selected according to ranging salinity, elevation and tree density. Other 11 plots which were sampled for the determination of HM contents were used to estimate parameters for the multiplier equation of pollutant (Equation (8)). The remaining 132 plots were used for model validation. Biomass data were taken from the work of Nam et al. [50].

Parameters were estimated by nonlinear regression techniques. This regression problem was solved by embedding an ODE (Ordinary differential equation) solver in the optimization toolbox of Matlab R2018b. For the estimation of parameters of biomass function and multiplier functions as shown in Equations (5)–(8), the regression problem was solved by standard nonlinear regression techniques (using Matlab R2018b).

### 2.5. Scenarios Setting

Scenarios modeling is a useful tool to understand the responses of a system to various factors and their combinations. In this study, we explored the trunk biomass of *R. apiculata* forest in the Can Gio area, which was given by Equation (5). Tree trunks represent the largest contribution to the total dry weight (DW) above-ground biomass. Three scenarios were set up:

(i) The baseline scenario (SC0) which assumed the unpolluted environmental condition. This scenario projects how the system may have developed within a four-decade period (1978–2020) under ideal conditions of no HM pollution.
(ii) Scenario based on actual chromium pollution (SC0P1) in the area in accordance with our observational data.
(iii) Worst case scenario assuming a twofold chromium load (SC0P2).

Tree densities in the forest stands were randomly generated by a uniform distribution in the range of 10–115 trees per plot (10 × 10 sq. meters) in accordance with our observational data.

The optimal number of scenarios is generally considered to be three or four [51]. The appropriate scenarios design should provide consistent outcomes that enable both the assessment of current conditions and assist future planning. We determined that comparing a baseline (no pollution) scenario with two clearly distinct scenarios of different HM levels would provide clear, useful, and visible alternatives for management planning.

**Figure 2:**
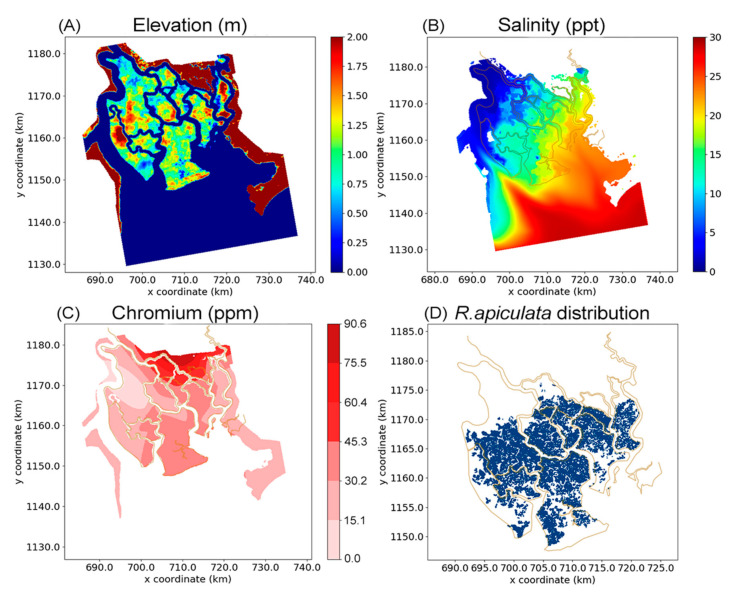
Spatial distribution of input data for the growth model, including an elevation map (**A**), a salinity map (**B**), a Cr content distribution map (**C**), and a map of *Rhizophora apiculata* forest (**D**).

In the simulation, the tree growth model was embedded into a life cycle model CGMM including the phases “Establishment–Growth–Competition–Reproduction–Mortality” (Anh [33]. Detailed explanation and equations for the life cycle are shown in Anh [33] Appendix C). Input data for the simulations include a topographic/bathymetric map of the study area, a map of salinity distribution and a map of root Cr content distribution. Note that we choose root concentrations of Cr as prediction variable rather than soil concentrations. The reason is that soil data reflect only the current environmental pollution state whereas root concentrations are the result of the HMs accumulation into the trees. Besides, a map of *R. apiculata* forest was also prepared. This map is available because this mangrove species was planted, and its data was well recorded. All these maps are shown in Figure 2 with a spatial resolution of 100 m × 100 m.

Chromium was chosen as the HM pollutant for our simulation because Cr is the main pollutant in our study area (in soil and in plant tissues). We also conducted experimental studies on the growth of young *R. apiculata* in dependence on Cr concentrations in combination with other factors [27,52]. Thus, data of Cr effect on plant growth are available to support the evaluation of simulation results. In our study, Cr oxidation forms were not specified; however, in general, both speciation forms Cr(III) and Cr(VI) occur in this area [31].

Other assumptions were: (1) All locations/plots used for simulations were fully forested with *R. apiculata*; (2) current mangroves at all sites were rehabilitated at the beginning and were naturally regenerated from seedlings of adult trees; (3) there were no disturbances following stand establishment. The simulation covers the time period from the year 1978 when *R. apiculata* forest was planted until the year 2020. The runs were initialized by a random uniform distribution of seedlings in each stand.

## 3. Results and Discussion

### 3.1. HMs Pollution in the Soil and Potential of HMs Retention by R. apiculata

#### 3.1.1. Soil HMs Pollution in the Area

Table 1 shows the HM data in soil, root, and leaf at the 11 sampling positions. The mean values of these HMs contents in the soil were 77.47 mg/kg for Cu, 9.82 mg/kg for Cr, and 5.87 mg/kg for Ni.

For conveniently describing the emerging polluted situation in the area, we divide the study area into two regions, the Thi Vai area and the Can Gio area (see Figure 1). Thi Vai catchment (Figure 1) is affected by the industrial activities on the East bank and the Can Gio area is directly influenced by activities from the center of Ho Chi Minh city (see Figure 1). The distribution of HMs contents among the different locations in the Can Gio (positions NP1, DK4, DK6, DK7, DK8, NP2) is more homogeneous than in the Thi Vai catchment (positions DN1, DN2, M5, M3, M1 in Figure 1). Standard deviation values of HM contents in the soil in Can Gio locations are smaller than those in the Thi Vai ones, they range from 0.28 to 6.29 for Can Gio and 2.48 to 29.79 for Thi Vai catchment (cf. Table 1). The HMs contents in the soils in the Thi Vai catchment are higher than those in the Can Gio, mean values for Cu, Cr and Ni respectively in the Thi Vai are 83.76, 16.35, and 9.30 mg/kg and in the Can Gio are 72.23, 4.38, and 3.01 mg/kg. Cu content is the highest among the three HMs both in Can Gio and in Thi Vai (the highest content is 134.03 mg/kg at the location DN2). Compared to our measurement campaign in 2013 [37], the pollution situation has generally improved. Chromium and Ni contents are lower than those in 2013 and their *EF* values are smaller than 1.5 (Table 2) falling in the *EF* class “not enriched”. But Cu (in 2018) in average is 3.2 times higher than this in 2013 (Cu content ranges from 60.4–134.03 mg/kg in 2018 and from 19.7–32.7 mg/kg in 2013 respectively, cf. Table 3). Its *EF* values are larger than 1.5 at all locations except DN1 (cf. Table 2) showing that Cu is moderately to highly enriched in the whole area. However, although concentrations of Cr and Ni below the limits for “enriched” (*EF* value < 1.5), they may not be related to low contamination levels as dilution effects may mask high HM rates of deposition (as was discussed by Costa-Böddeker et al. [38]).

The HM pollution in this region has happened for about two decades since ~2000. The Thi Vai river and its surroundings have been severely polluted because of the direct discharge of wastewater from the industrial companies. Costa-Boddeker et al. [32] analyzed the sediment cores in this region and reported that the violations of HM pollution especially for Cu, Cr, and Ni were observed particularly after the establishment of the industries in the area in the late-1990. Concentrations of these HMs from 2003 to 2012 ranged from 107.70–208.80 mg/kg for Cr, 26.74–82.32 mg/kg for Cu, and 56.25–82.99 for Ni respectively in the upstream section. These concentrations were lower in the downstream section with values range from 51.6–82.5 mg/kg for Cr, 11.5–38.3 mg/kg for Cu, and 24.7–46.5 mg/kg for Ni respectively (Costa-Boddeker et al. [32], see Table 3). These HMs contents are in consist with the increment number of industrial companies in this catchment. The number of industrial companies in the Thi Vai catchment increased from 10 companies in 2002 to 135 companies in 2017 (data from the Ho Chi Minh City Department of Natural Resources and Environment) with main activities in tannery, mechanical engineering, electrical engineering, packaging, textile and dyes industry, oil activities and cement production. Until 2006, over 60% of the industrial zones had not implemented an adequate wastewater treatment system and the untreated wastewater was discharged directly into the river [31].

For the Can Gio area, Costa-Böddeker et al. [38] reported the increasing trend of HMs pollution was from ~2002. Chromium, Cu and Ni were also the highest concentrations in sediments ranging from 27.1 μg/g to 71.5 μg/g for Cr, 7.1 μg/g to 27.0 μg/g for Cu, and 11.7 μg/g to 56. 3 μg/g for Ni (Table 3). Beside those HMs, the area has also been polluted by Pb and Co with the order Cr > Ni > Cu > Pb > Co [38]. Following Costa-Böddeker et al. [38], enhanced HM concentrations in the sediment of the Can Gio area may have originated from both natural and anthropogenic sources, with the latter being the main contributor in recent years. The anthropogenic sources are mostly associated with the industrialization process in Southern Vietnam, the impact of shrimp farms as well as oil spills and untreated wastewater. Factors such as shoreline erosion caused by tides and deforestation may also have contributed to these HMs distribution [38].

HM pollution occurring in the soils of mangrove areas worldwide are mostly attributed to industrial activities. Compared to other mangrove regions, the contents of HMs associated mostly with anthropogenic sources in our region are lower than in some other industrialized mangrove regions, see for instance the Maipo mangrove area in Hong Kong or Surabaya in Indonesia and Yanbu in Saudi Arabia (Table 3). However, Cu contents are higher than in other contaminated areas (with values from 1.2 to 4-fold higher, Table 3), particularly within the sites upstream in Can Gio (NP1, DK4) and downstream in Thi Vai catchment (DN2, M3, M1). The intensified industrialization over the past two decades has brought about the moderate to high enrichment of the HMs Cu, Cr and Ni. It was said that the complex interplay of the dynamics of sediments such as increasing erosion, shoreline retreat and transportation of HMs may be hiding the real state of HM pollution and ecological disturbances in this region [32].

#### 3.1.2. Potential of the HMs Retention by the Mangrove Species *R. apiculata*

In the tissues of *R. apiculata*, the concentrations of Cr, Cu, and Ni in the root and the leaf are shown in Table 1. In the root, the mean values of Cr, Cu, and Ni are 2.63, 2.52, and 2.01 mg/kg respectively, ranging from 0.26 to 11.69 mg/kg for Cr, 0.21 to 8.17 mg/kg for Cu and 0.11 to 8.54 mg/kg for Ni. In the leaf, the average contents of Cr, Cu, and Ni are 0.83, 2.1 and 0.39 mg/kg respectively, ranging from 0.23 to 2.05 mg/kg for Cr, 0.18 to 7.83 mg/kg for Cu and 0.005 to 1.81 mg/kg for Ni. Among the positions, Cu contents in tree tissues (root and leaf) at locations along the Thi Vai river (locations DN1, DN2, M5, M3, M1) are higher than those in the Can Gio (NP1, DK4, DK6, DK7, DK8, NP2). Even though the concentration of Cu in the soil is high compared to this measured in 2013 (Table 3), this content is low in plant tissues both in leaf and root compared to the tissue values measured in 2013. This could be explained by the incident time of HM. Copper might have just recently entered the environment. Similar to what was found in the soil, the contents of HMs are nearly homogeneous for all locations in the Can Gio but not for the Thi Vai region (Table 1, standard deviation ranges from 0.15 to 0.41 for HMs in the root and 0.19 to 0.56 for HMs in the leaf in the Can Gio area, respectively; while this value ranges from 2.01 to 4.03 for HMs in the root and from 0.66 to 2.16 for HMs in the leaf respectively in the Thi Vai catchment).

Average Cr concentration in the root of *R. apiculata* is the highest concentration among the three HMs in the root, but this value is lower than in the roots of other mangrove species of other regions (see Table 3) except in some mangrove roots in Maipo, Hong Kong (~2 times higher). While in the leaf, Cu concentration is the highest among the three HMs. However, these three HM concentrations in our leaves are still lower than in the leaves in other mangrove regions as shown in Table 3.

The HMs accumulated in root and leaf tissues clearly demonstrate the potential of *R. apiculata* in the retention of HM. Because these HM contaminants in the soil occurred since ~2003, the trees accumulated high concentrations of these soil enriched HMs in their tissues. It was found in our previous work Nguyen et al. [37] that the plant tissues at 11 sampling positions (the same positions as in this work and is shown in Figure 1) accumulated high amounts of Cr, Ni, and Cu especially in the tree cores [37]. Tree stems include the sapwood which engages in transport of water and minerals to the crown of the tree. The sapwood is periodically converted to heartwood (in the tree cores) which constitutes the layers of death sapwood cells. Therefore, by time, plants accumulate elements and immobilize them in their dead cells in the heartwood (in tree cores). The high contents of these soil contaminants in the tree cores corroborate the assumption that these elements have been accumulated since a long time.

The HMs retention by mangroves has been reported in many countries. Mangrove wetlands are often considered as sinks for contaminants. There are many cases in which wetland plants (mangroves) are utilized for removal of pollutants, including metals. This functionality of mangroves has been studied and proven to be a good practical method to treat pollutants. The study of Kamaruzzaman et al. [4] with *R. apiculata* in Malaysia corroborated the potential of metal accumulation especially in their root. MacFarlane et al. [5] conducted experiments and reported that Cu was accumulated in root tissues of *Avicennia marina* under a certain concentration limit (200 µg/g). Nazli and Hashim [6] found the accumulation of Cu and Pb in roots and leaves of *Sonneratia caseolaris* and concluded that the roots of this species have high capacity in taking up heavy metals and could be a potential phytoremediation species for heavy metal treatment in Malaysian mangrove ecosystems. Chowdhury et al. [7] concluded that the species *Sonneratia apetala* and *Avicennia officinalis* are potential hyperaccumulators which accumulated high amounts of Cu (25.89 mg/kg), Fe (1376.7 mg/kg), and Cr (2.85 mg/kg) in the pneumatophore. Fengzhong et al. [8] found that *Avicennia marina* has a high removal ability for lead (Pb, 83.8%) and Cd (74.2%) and that *K. candel* has ability to absorb copper (Cu, 70.5%) and nickel (Ni, 50.5%). Wen-jiao et al. [9] found that *Rhizophora stylosa* in Yinglou bay (China) absorbed high amounts of cadmium (Cd). Titah and Pratikno [10] discussed that *Avicennia alba* can be considered for use in phyto-monitoring and phytoremediation of Cr in coastal areas. In general, these authors stressed the potential role of mangroves in HM retention. Thus, there is no doubt that mangroves in general and *R. apiculata* in our study area possess a remarkable capacity to retain HMs and tolerate relatively high levels of metals.

Mangrove plants develop a series of enzymatic and nonenzymatic antioxidants to scavenge reactive oxygen species (ROS) and reduce the toxic effects of abiotic stresses, including HMs [56]. Field inventories and laboratory experiments showed that the mangrove trapping of HMs was a very efficient and fast phenomenon [56,57]. The tolerance of mangrove plants to HM stress is normally a mixture of metal avoidance and scavenging of reactive oxygen species (ROS) [56].

Mangrove soil with the anoxic nature, usually with negative redox potential and high amounts of sulfide, iron, and organic matter, decrease the metal solubility and bioavailability that makes sediment be a long-term sink [56,58,59]. Mangrove sediments generally have a high capacity for adsorbing and holding trace metals, however, excess loading of HMs may exceed the binding capacity of the sediment [56,60]. Salinity is an important variable for the distribution and absorption of HM in the soil [31,32]. It has a key role to control the bioavailability and toxicity of HM in sediments. Changes in salinity may cause the mangrove sediments to lose their metal-binding capacity, resulting in mobilization of metals [56]. The competition between major cations and HM for sorption sites is enhanced with increasing salinity [61]. The mangrove sediments then shift from a HM sink to a HM source, and such disruption is often associated with human activities [56,58].

Among the tree organs, root of mangrove tree has an important role in depurating water and sediment by retaining metals. Metals are generally highly bioavailable and accumulated in mangrove roots. The common studied HMs, such as Cu, Zn, Cd, Cr, and Hg, generally showed high bioconcentration factors in roots. Thus, under increasing HM pollution pressure from the rapid industrialization and urbanization, it is said that mangroves can serve as a natural wastewater treatment system, which could be considered as a phytoremediation process.

### 3.2. Growth of R. apiculata in Different Environmental Conditions

#### 3.2.1. Tree Growth Rate (*G*) and Influencing Factors

The structure of mangrove forests, their health and their ecosystem functions are strongly influenced by the factors resources (nutrients), regulators (soil salinity, pollutants), and hydro-regimes (duration, frequency, and depth of inundation) and their mutual interactions [17,18,19,20,21]. The long-term influence of these driving factors on tree development is in a way summarized by the growth rate *G*. The data of *G* values can be extracted by inserting the growth parameters (208 data plots) into Equation (9).

The growth rate (*G*) extracted from 76 plots among the 208 data plots were used to estimate parameters for the multiplier Equations (6)–(8) which expressed the influences of above driving factors. Table 4 shows the estimated parameter values.

**Figure 3:**
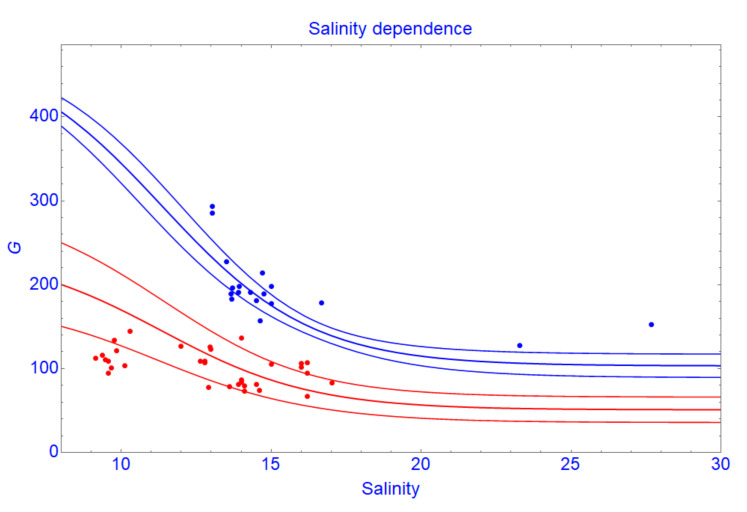
Growth rate of the trees in the plots which are located on large salinity gradients and have the same range of tree density. The red points and curves are the data plots and the section and 95% confidence bands constructed at a density of 80 trees per plot, respectively. The blue points and curves are the data plots and the section and 95% confidence bands constructed at a density of 15 trees per plot, respectively.

The extracted tree growth rate (*G*) from the data shows a negative correlation (r = −0.78) with the pore water salinity. Figure 3 shows growth rates of trees in the plots located on large salinity gradients and have similar tree density. Along the salinity gradient, growth rates (*G*) of trees decrease. This effect is enhanced at high tree density. This relationship agrees with other previous studies on the impacts of salinity on the growth of mangroves [62,63]. Reef et al. [64] supposed that nutrient availability of mangroves is controlled by the redox state of the soil, which may be influenced by salinity. Dai et al. [65] reported that salt stress influences mangrove productivity. A common response to salt stress is that the leaf surface expansion is reduced, thereby reducing photosynthesis, which in turn reduces carbohydrate production [66,67,68]. Experiments from our previous study [69] on young *R. apiculata* in this area showed that plants growing on salt-leached soil have the highest growth rates and on natural saline soil have the lowest growth rates. Salinity in the natural saline soil can be considered as a toxic agent influencing the uptake of nutrients and thus plant growth. This might be due to competitive inhibition of ammonium uptake by sodium. Thus, in high salinity, growth rate (*G*) of mangrove trees decreases.

Beside the physical environment, tree density also affects tree growth since the trees compete to each other for nutrient and light. Tree densities decline with forest age, notably because of the silvicultural practice of intermediary thinning for removing competing dominant trees [70]. However, tree density also declined in natural forests due to physiological tolerances and competitive interactions [71]. 

**Figure 4:**
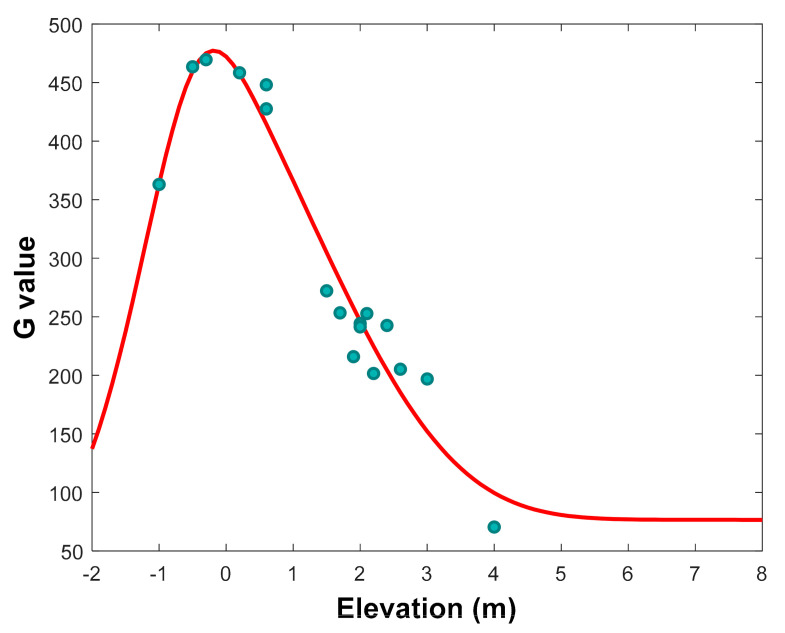
Fitted curve of model growth rate *G* (the red curve) of *R. apiculata* and the *G* values extracted from data (the round points) along elevation gradient.

Another factor which directly influences mangrove growth is the inundation. Inundation stress and increasing sea level are influencing the growth of mangroves. These factors are reflected by the ground elevation. The higher water level provokes changes of elevation gradients. The main consequences are the decline of biomass, tree density, basal area, and C pool. Figure 4 shows the fitted curve of model growth rate *G* of *R. apiculata* and the *G* extracted from data. The large gradient of elevation influences the growth of mangroves such as those observed in our samples. This result shows that *R. apiculata* develops well within elevation range from −0.5 to 0.5 m. Gilman et al. [72] noted that mangrove species maintain their preferred hydroperiod, they may also expand laterally into areas of suitable elevation which are the environmental conditions for recruitment and establishment of mangroves in new areas. Schwarzer et al. [39] argued that any loss in elevation makes mangroves more susceptible to submergence from sea-level rise. Ellison and Farnsworth [73] reported that mangroves need to grow in a stable condition.

#### 3.2.2. Tree Growth Rate (*G*) in Polluted Condition

The data of growth parameters (mean *dbh*, mean height *H*, age, mean growth rate *G*) and the environmental factors (soil salinity and *EF* values for Cr, Cu, and Ni in the soil) at the 11 measured forest stands are shown in Table 2. These growth parameters reflect the prevailing environmental conditions during the lifetime of a tree. The highly contaminated stands (Table 2, positions DK6, DK7, DK8, NP2, and M1 with *EF* (Cu) value > 2.5) have *G* values lower than the other stands. The lowest value of *G* appears at stand M1, where trees grow on interactive negative conditions of high salinity (17.1 ppt), high density (17 trees) and highly contaminated soil (*EF* (Cu) = 3.9, Table 2).

**Figure 5:**
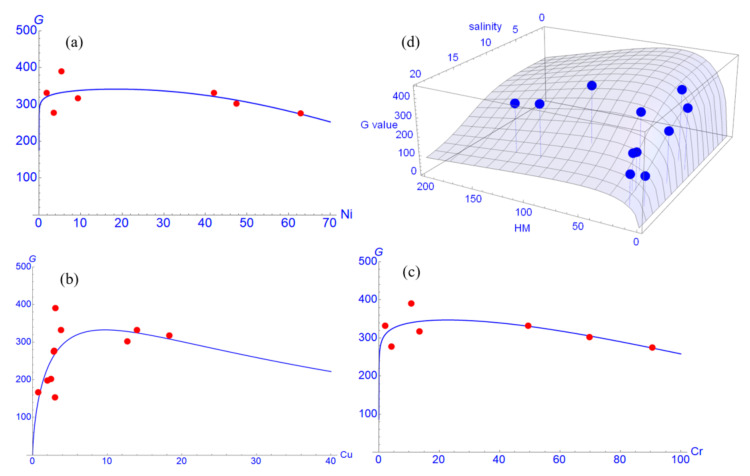
Tree growth rate (*G*) is dependent on concentration (mg/kg) of the contaminants Ni (**a**), Cu (**b**), and Cr (**c**). This is a fit to the multiplier function for metal contaminants (Equation (8)). (**d**) Response surface of *G* in dependence on the total root concentration of Ni, Cu, and Cr and on the soil salinity.

We also found that the HMs Cu, Cr, and Ni accumulated in plant roots negatively affect the plant growth at high concentration levels (Figure 5). These results suggest that mangrove growth is limited by the excessive presence of HMs which are taken up by the roots. Our data indicate that in average, Cr, Cu, and Ni concentrations accumulated in root over a concentration of 20 mg/kg are negatively correlated with growth of *R. apiculata* as expressed in Figure 5a–c. However, the lower *G* values at low concentrations may hint to a hormesis effect. Homesis effects of the heavy metals Pb, Cu and Cd were found in the experiments of Zhang et al. [74] with *Sonneratia apetala*. Unfortunately, the duration of their experiment was not long enough to observe fully plant behavior. The interactive effect of these HMs accumulated in tree root on tree growth rate G in relation to the soil salinity is also shown in Figure 5d.

For Cu, MacFarlane and Burchett [75] reported that *Avicennia marina* was found to be highly tolerant to Cu. It was accumulated in root tissue but at concentrations of 200 μg/g and higher, no further increases in root Cu levels occurred. Significant reductions in seedling height, leaf number and area were found with significant increases in Cu concentrations at 100 μg/g sediment Cu. At Cu sediment levels of 400 μg/g, a decrease in total biomass and root growth inhibition was observed. Chawla and Patel [76] observed changes in chlorophyll contents (total chlorophyll and ratio chl a/b were examined) by the application of different concentrations of Cu in seedlings of the mangrove species *Avicennia marina* (Forsk.)Vierh. The results indicated that the content of total chlorophyll increased at low levels of Cu but started declining at higher concentrations.

For Cr, our previous experiments in Richter et al. [27] and Nguyen et al. [69] showed that the phytoremediation performance of young *R. apiculata* in treating Cr was at the maximum Cr concentration at 600 mg/L in industrial wastewater. Oliveira [77] discussed that is Cr structurally similar to other essential elements and may therefore displace the nutrients from physiological binding sites.

For Ni, to our knowledge, there have been limiting studies on effects of excess Ni on mangrove growth. Nickel and Cu as micronutrients for plants play a role as brokers of redox transformations [78]. However, Ahmad and Ashraf [79] discussed that although Ni is essential to several plant metabolic phenomena, it is extremely toxic to plants when present at excessive levels in the soil or in nutrient solutions to which plants are exposed. The general signs associated with Ni toxicity in plants include reduced shoot and root growth, poor development of the branching system, and deformation of various plant parts.

### 3.3. Simulation Results

#### 3.3.1. Model Validation

**Figure 6:**
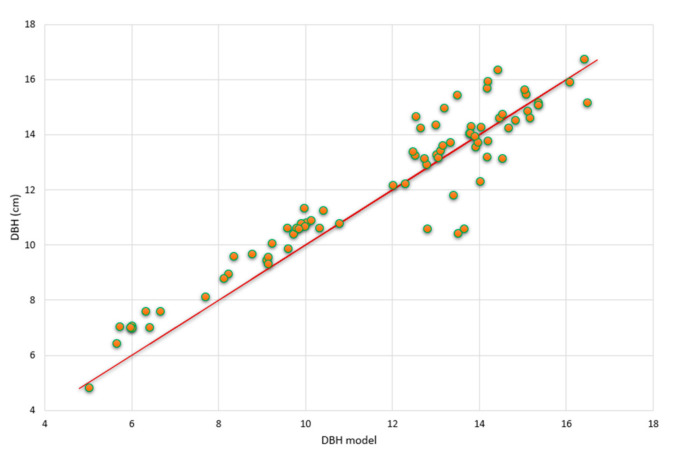
Measured *dbh* values vs. model predictions for the validation data. The coefficient of variation value is R^2^ = 0.87.

**Figure 7:**
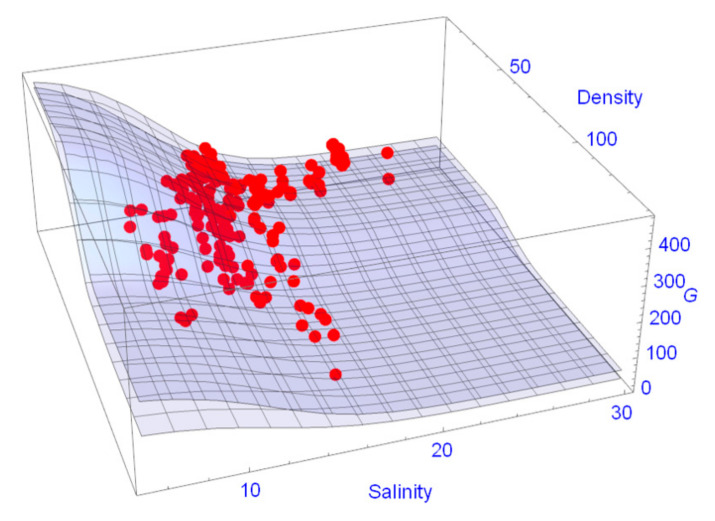
95% confidence surfaces together with the *G* values of the validation data set.

As already mentioned in Section 2.4.2, the data set was split into one data set for parameter estimation (76 plots) and one for validation (132 plots). In Figure 6 the *dbh*s of *R. apiculata* predicted from the model are plotted against the measured *dbh*s. There are no apparent systematic deviations between data and model predictions and the correlation coefficient is R^2^ = 0.87. The predictions of *G* values in dependence of the variables’ density and salinity together with the 95% confidence surface are shown in Figure 7. The coefficient of variation is R^2^ = 0.8902. One clearly recognizes the nonlinearity of the response surface both with respect to density and salinity (Figure 7).

It should be emphasized that the role of mono specific forest plantation here is especially important as a long-term experiment allowing the assessment of tree development of a single species in different environments. The validation results justify the application of the model for predicting the production of mangrove trees at each age in dependence on different environmental gradients and HM pollution loads. The model can thus be employed as a decision support tool for the management of mangrove forest.

#### 3.3.2. Scenarios Simulation Results

**Figure 8:**
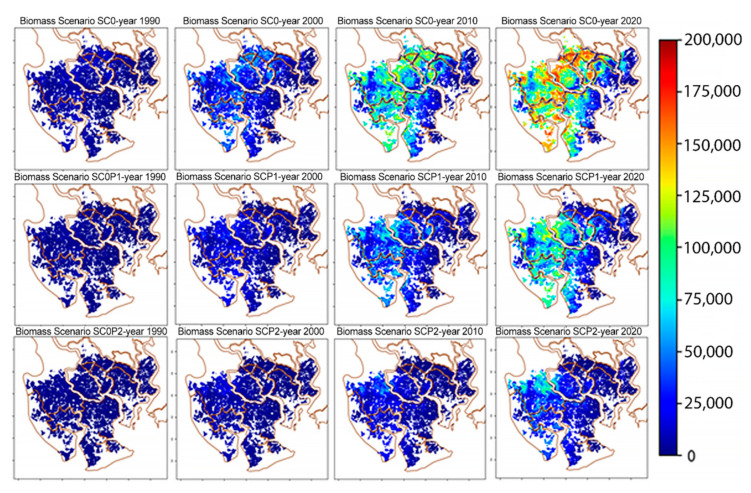
Distribution patterns of trunk biomass over time in different scenarios. The columns show the biomasses at different times, the rows show the results for the three scenarios.

**Figure 9:**
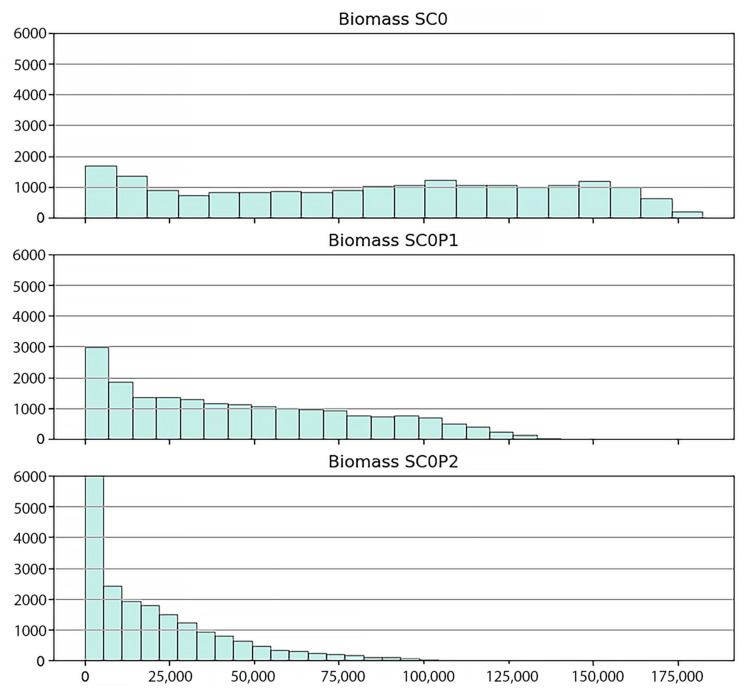
Biomass (kg/ha) histogram of three scenarios in the last phase of simulations (in 2020).

Figure 8 shows snapshots of the spatial distribution of biomasses at four time points for the three scenarios described above. The histograms of biomasses at the final stage of simulations obtained for the three scenarios are given in Figure 9. The histograms are shifting to the left towards smaller biomass values with growing pollution levels and have lower variances.

Under the baseline scenario a rather inhomogeneous biomass distribution with large variation is obtained reflecting the unpolluted natural environmental conditions. Biomasses range from 5000 (~1800 stands) to 175,000 (~100 stands) kg/ha in 2020. Under the presence of HM, the range of biomasses becomes smaller and the distribution becomes more homogeneous (3000 stands and 6000 stands with trunk biomass 5000 kg/ha for SC0P1 and SC0P2, respectively). The occurrence of stands with high biomass are very limited (90 stands have the maximal biomass 125,000 kg/ha in SC0P1 and ~100 stands have the biomass >25,000 kg/ha for SC0P2, Figure 9).

The biomasses obtained from the simulations compare well with empirical data of other studies. In the work of Vinh et al. [80] the average measured trunk dry biomass of *R. apiculata* in Can Gio was reported 76,250 kg/ha (76.25 ± 5.13 tons/ha). It resulted from dry weighing 36 felled trees with ranging diameters from 7–36.2 cm at one specific site. In the study of Hoan [81] with 45 felled trees in different locations, the average trunk dry biomass of the *R. apiculata* was in the range of 47,600 to 139,800 kg/ha with diameters ranging from 9 to 21 cm. Maximum trunk biomasses in 2020 obtained from our simulations are 175,000 kg/ha in the baseline scenario (ideal environmental condition), 140,000 kg/ha and 100,000 kg/ha in the SCOP1 and SC0P2 scenarios respectively (cf. Figure 9). The slight difference in our predicted biomass compared to the data from the above authors are probably due to the large variation in tree size. In our study, biomass was obtained from the summation over all trees in one-hectare plots regardless of tree size (there exist young trees to adult trees), while in the referenced work above biomass was weighed based on the adult trees with starting diameter of 7 cm.

By our analysis of field data and simulation studies we have assessed the influence of HMs and other factors tree density, soil salinity and ground elevation on the trunk biomass of *R. apiculata*. Variation of trunk biomass of *R. apiculata* forest is much higher among the plots in SC0 with no HM pollution. This suggests that the interactions of soil condition and tree density with HM pollutants were responsible for the slower growth and for the smaller biomass in the two HM polluted scenarios (SC0P1 and SC0P2).

The simulations show the order of magnitudes involved in the reduction of biomass due to HM pollution. Total trunk biomasses in 2020 of trees growing under HM stress since 1978 amount to 850,000 tons and 350,000 tons for the SC0P1 and SC0P2 respectively, in contrast to 1,750,000 tons achieved under no HM stress conditions (for the SC0). The tree growth in the SC0 is influenced just by the natural factors of salinity, elevation, and tree density. These natural environmental factors have changed only gradually by the complex dynamics of the estuarine environment, so we can consider these factors quasi as stable environmental conditions (without considering the effects of climate change). Thus, to achieve mangrove stands with high biomass, we should control the pollution by reducing the amount of HMs release.

### 3.4. Toward a Balancing Management Approach on Using Mangroves to Clean Up Polluted Environment and to Protect the Mangrove Ecosystem

The Ministry of Agriculture and Rural Development (MARD) in Vietnam reported five main causes of the Vietnamese mangrove loss: (1) Non-sustainable use of mangrove ecosystems for aquaculture, (2) storms and natural disasters, (3) deforestation of mangrove forests for timber and natural resources, (4) multi-source pollutants from agriculture, industrial and urban areas, and (5) lack of sufficient regulatory mechanisms for the protection and sustainable development of mangrove ecosystems. Mangrove plants show high metal retention abilities. Because of this ability, mangroves have been proposed for utilizing for the removal of pollutants, including metals. The problem is, on the one hand, mangroves have an important role and function as a natural phytoremediation system; however, on the other hand mangrove ecosystems themselves are highly sensitive to all kinds of disturbances.

The high rate of heavy metal contamination in mangrove areas has become a global problem owing to its high toxic ability, abundance, and persistence [2,31,45]. However, approaches set up for monitoring and evaluating components are often absent, or methods adopted are mostly inconsistent between projects [82,83,84]. This reveals the significant lack of current agreement on holistic ecosystem health monitoring. There is therefore a pressing need for innovative approaches to improve the management of the remaining mangroves by developing a reliable and thorough assessment method for determining their ecosystem status and their likely future trajectory.

The simulation model presented in this paper is a first step towards the support of a balancing management approach involving both the phytoremediation role of mangroves by absorption of HM and its vulnerability by HM. The model is capable of analyzing quantitatively the tradeoff between pollutant removal and adverse effects on mangrove growth.

Unhealthy vegetation leads to the breakdown of important functions of the mangrove wetlands. The next step in the development of a decision support system is the use of remotely sensed data as the input for mangrove dynamic models (such as this of our model). Such consideration is not now a routine part of mangrove management planning and rehabilitation processes. While field work is more challenging in mangrove habitat, remotely sensed data is capable of mapping mangrove distribution, estimating biophysical parameters and characterizing ecosystem process. The combination of remotely sensed data and growth models can open the way to a data-and-model driven management of mangrove forest stability for coastal protection.

## 4. Conclusions

We have assessed the effects and interactions of HM pollutants and other environmental stressors on trunk biomass of *R. apiculata* forest in the Can Gio Mangrove forest and have shown that the tradeoff between using mangroves as a natural phytoremediation system and reserving this highly sensitive ecosystem is a challenge. It should be emphasized that the role of mono specific forest plantation here is particularly important as a long-term experiment allowing the assessment of tree development of a single species in different environments with well documented age of the stands. Data acquisition in natural forests with unknown tree ages demands much more efforts concerning the design of appropriate sampling schemes.

Our result may support mangrove ecosystem managers in their difficult task to protect this vulnerable ecosystem. In order to avoid further negative effects for the mangrove ecosystem of this protected area, the pollution load must be decreased in the future. Further studies are required to advance the understanding of the long-term trends and the complex interplay between contaminant concentrations, hydrodynamics, anthropogenic stressors and the mangrove ecosystem. Also, adequate solutions for management policies in cooperation with stakeholders from industry, administration, environmental protection and forestry based on a data-model driven network of monitoring systems need to be developed.

## Figures and Tables

**Table 1:** Contents (value, max value, mean value, min value and standard deviation std) of the heavy metals Cu, Cr, and Ni (mg/kg) in soil and in tissues of the mangrove species *R. apiculata* at the 11 sampling positions.

			SOIL			ROOT			LEAF		
			Cu	Cr	Ni	Cu	Cr	Ni	Cu	Cr	Ni
CG	*Upstream*	**NP1**	80.04	4.96	3.31	0.21	1.06	0.57	0.31	1.51	0.71
		**DK4**	70.82	3.76	2.72	0.60	0.70	0.45	0.72	0.37	0.12
		**DK6**	64.51	4.35	2.63	0.31	0.32	0.11	0.25	0.29	0.01
		**DK7**	65.65	4.44	3.08	0.54	0.26	0.17	0.39	0.40	0.09
		**DK8**	75.76	4.28	3.25	0.37	1.20	0.66	0.18	0.23	0.01
	*Downstream*	**NP2**	76.62	4.49	3.06	0.47	0.32	0.22	0.36	1.27	0.46
TV	*Upstream*	**DN1**	62.41	12.39	8.49	3.07	4.46	4.90	3.53	0.39	0.14
		**DN2**	134.03	19.84	11.35	8.17	11.69	8.54	2.12	0.49	0.01
		**M5**	60.40	21.14	12.01	5.55	2.19	2.19	4.03	1.04	0.27
		**M3**	79.46	13.62	8.82	4.88	1.52	1.30	3.40	2.05	1.82
	*Downstream*	**M1**	82.51	14.75	5.82	3.56	5.24	3.08	7.83	1.08	0.76
CG		**max**	80.04	4.96	3.31	0.60	1.20	0.66	0.72	1.51	0.71
		**mean**	72.23	4.38	3.01	0.42	0.64	0.36	0.37	0.68	0.23
		**min**	64.51	3.76	2.63	0.21	0.26	0.11	0.18	0.23	0.01
		**std**	6.29	0.39	0.28	0.15	0.41	0.23	0.19	0.56	0.29
TV		**max**	134.03	21.14	12.01	8.17	11.69	8.54	7.83	2.05	1.82
		**mean**	83.76	16.35	9.30	5.05	5.02	4.00	4.18	1.01	0.60
		**min**	82.51	14.75	5.82	3.56	5.24	3.08	7.83	1.08	0.76
		**std**	29.79	3.90	2.48	2.01	4.03	2.87	2.16	0.66	0.74

Note: bold: to press the ID of the positions.

**Table 2:** Values of tree growth parameters and environmental factors at the 11 measurement plots.

Position			Mean *dbh* (cm)	Mean Height (cm)	Density/Plot (10 × 10 sq·m)	Age (Year)	Porosity Salinity (ppt)	Mean Tree Growth Rate (*G*)	*EF* (Cu)	*EF* (Cr)	*EF* (Ni)
**Can Gio**	**NP1**	***Upstream***	18.62	1989.97	26	26	8.0	390.53	***3.41***	0.11	0.09
	**DK4**		10.46	1232.91	42	32	12.4	277.08	***4.13***	0.11	0.10
	**DK6**		22.29	2293.78	8	38	12.9	275.46	***2.53***	0.09	0.07
	**DK7**		15.06	1673.72	13	37	15.5	201.61	***2.42***	0.08	0.08
	**DK8**		19.71	2082.55	11	40	15.5	197.74	***2.62***	0.07	0.07
	**NP2**	***Downstream***	13.14	1501.13	16	39	18.2	166.91	***3.29***	0.10	0.09
**Thi Vai**	**DN1**	***Upstream***	16.13	1770.92	13	25	8.0	332.13	*1.18*	0.12	0.11
	**DN2**		16.16	1757.51	20	25	9.6	331.82	***3.49***	0.26	0.20
	**M5**		13.99	1574.53	11	31	11.3	317.36	***1.83***	0.32	0.24
	**M3**		11.97	1381.85	20	23	13.5	300.98	***2.53***	0.22	0.19
	**M1**	***Downstream***	12.45	1428.23	17	32	17.1	152.97	***3.90***	0.35	0.18

Note: bold italic numbers are the *EF* values larger than 1.5.

**Table 3:** Range of heavy metal concentrations in the soil, mangroves leaf and root of the Can Gio Mangrove Forest and of the other mangroves worldwide.

Location	*Species*	Part	Cr (mg/kg)	Cu (mg/kg)	Ni (mg/kg)	Reference
**This study, Can Gio, Vietnam (2018)**	Soil	Soil	**3.76–21.14**	**60.4–134.03**	**2.63–12.01**	**This study**
	*Rhizophora apiculata*	Leaves	**0.23–2.05**	**0.18–7.83**	**0.005–1.82**	
		Roots	**0.26–11.7**	**0.21–8.2**	**0.11–8.54**	
Can Gio, Vietnam (2013)	**Soil**	Soil	86–241	19.7–32.7	56.6–247	[37]
	***Rhizophora apiculata***	Leaves	1.48–23.6	2.77–6.41	1.4–25.7	
		Roots	2.14–90.6	0.746–18.3	1.79–62.9	
Can Gio, Vietnam (2002–2012)	**Sediment core**	Sediment	107.70–208.80	26.74–82.32	56.25–82.99	[32] Note: Thi Vai side, upstream
Can Gio, Vietnam (2002–2012)	**Sediment core**	Sediment	51.6–82.5	11.5–38.3	24.7–46.5	[32] Note: Thi Vai side, downstream
Can Gio, Vietnam (1977–2011)	**Sediment core**	Sediment	27.1–71.5	7.1–27.0	11.7–56.3	[38] Note: Can Gio side
Mai po, Hong Kong	**Soil**	Soil	20–74.6	51.1–87.4	43.9–86.9	[53]
	***Acanthus ilicifolius***	Roots	1.6–6.2	25.2–65.4	8–20.1	
	***Aegicerus corniculatum***	Roots	1.8–5.8	22.4–46.2	6–32.01	
	***Kandelia candel***	Roots	1.8–6.4	19.6–29.4	4–28.001	
Surabaya, Indonesia	**Soil**	Soil	47–79.3			[10,54]
	***Avicennia alba***	Roots	25.4–55.3			
	***Avicennia marina***	Roots	28–92.25			
Yanbu, Red Sea, Saudi Arabia	**Soil**	Soil	14.9–289	17.2–217.2	27.3–241.8	[55]
	***Avicennia marina***	Leaves	14.2–50.1	18.1–40.2	16.1–56.3	
		Roots	16.3–40.5	16.8–37.3	17.2–38.2	

Note: bold and italic text and numbers are values of this study.

**Table 4:** Result of the parameter estimation.

	Parameter	Value			
Density multiplier	$a0_{1}$	0.402741			
	$d_{1}$	0.156347			
	$tr_{1}$ (trees/100 m^2^)	66.9241			
Salinity multiplier	$a0_{2}$	0.21276			
	$d_{2}$	−0.4			
	$tr_{2}$ (ppt)	11.348			
Elevation multiplier	$a_{1e}$	0.027			
	$el_{1}$	−0.96			
	α	13.1			
	$el_{2}$	1.48			
	β	6.02			
	$a_{2e}$	0.11			
Pollutant multiplier			Cu	Cr	Ni
	$th_{1}$		3.28	1.89	0.53
	$th_{2}$		54.47	170.25	105.43
	α1		0.68	0.12	0.06
	α2		0.81	1.69	2.53
